# Public perspectives on acquired brain injury rehabilitation and components of care: A Citizens’ Jury

**DOI:** 10.1111/hex.13176

**Published:** 2020-12-02

**Authors:** Natasha A. Lannin, Megan Coulter, Kate Laver, Nerida Hyett, Julie Ratcliffe, Anne E. Holland, Libby Callaway, Coralie English, Peter Bragge, Sophie Hill, Carolyn A. Unsworth

**Affiliations:** ^1^ Department of Neuroscience Central Clinical School Monash University Clayton Vic. Australia; ^2^ Occupational Therapy Department Alfred Health Melbourne Vic. Australia; ^3^ Flinders University Adelaide SA Australia; ^4^ La Trobe Rural Health School La Trobe University Melbourne Vic. Australia; ^5^ College of Nursing and Health Sciences Flinders University Adelaide SA Australia; ^6^ Central Clinical School Monash University Clayton Vic. Australia; ^7^ Physiotherapy Department Alfred Health Melbourne Vic. Australia; ^8^ Monash University Clayton Vic. Australia; ^9^ School of Health Sciences Priority Research Centre for Stroke and Brain Injury University of Newcastle Callaghan NSW Australia; ^10^ BehaviourWorks Australia Monash Sustainable Development Institute Monash University Clayton Vic. Australia; ^11^ Centre for Health Communication and Participation and School of Psychology and Public Health La Trobe University Melbourne Vic. Australia; ^12^ School of Health Federation University Churchill Vic. Australia; ^13^ Department of Rehabilitation Jonkoping University Jonkoping Sweden

**Keywords:** consumer participation, decision making, deliberative methods, health policy, traumatic brain injury

## Abstract

**Background:**

Brain injury rehabilitation is an expensive and long‐term endeavour. Very little published information or debate has underpinned policy for service delivery in Australia. Within the context of finite health budgets and the challenges associated with providing optimal care to persons with brain injuries, members of the public were asked ‘What considerations are important to include in a model of care of brain injury rehabilitation?’

**Methods:**

Qualitative study using the Citizen Jury method of participatory research. Twelve adult jurors from the community and seven witnesses participated including a health services funding model expert, peak body representative with lived experience of brain injury, carer of a person with a brain injury, and brain injury rehabilitation specialists. Witnesses were cross‐examined by jurors over two days.

**Results:**

Key themes related to the need for a model of rehabilitation to: be consumer‐focused and supporting the retention of hope; be long‐term; provide equitable access to services irrespective of funding source; be inclusive of family; provide advocacy; raise public awareness; and be delivered by experts in a suitable environment. A set of eight recommendations were made.

**Conclusion:**

Instigating the recommendations made requires careful consideration of the need for new models of care with flexible services; family involvement; recruitment and retention of highly skilled staff; and providing consumer‐focused services that prepare individuals and their carers for the long term.

**Patient and public contribution:**

As jury members, the public deliberated information provided by expert witnesses (including a person with a head injury) and wrote the key recommendations.

## 1 INTRODUCTION

Designing rehabilitation services for people with a moderate to severe brain injury is complex due to the variability in case presentation, goals, and medical and functional needs. The lifelong nature of brain injury, as well as costs associated with long‐term care, demands that policymakers and health‐care organizations use the most effective and efficient methods to organize patient care. Policymakers, however, struggle to appreciate the complexity of the medical, functional, social and financial circumstances that accompany people who have a brain injury and rarely achieve consensus on the nature and length of services required.^1^, ^2^ Traditionally, policy decisions about brain jury rehabilitation are driven by objectives to continue service provision in its current form, by research evidence or by the views of individual clinicians. Policymakers view clinicians as providing expert, unbiased and objective guidance in this area.^3^ There is also a growing evidence base for including patients and their families in health‐care decision making,^4^ with a number of models and methods of engaging consumers being reported together with their relative merits and limitations.^5^, ^6^ In contrast, resource allocation discussions have rarely included citizens.^7^, ^8^, ^9^ It is thought that evidence provided by citizens is subjective and biased and that citizens have limited capacity to contribute relevant knowledge^10^ to health policy debates. Therefore, there is very limited evidence of citizens’ information being used by policymakers for resource allocation and policy development.^4^, ^11^ However, given the significant tax‐payer investment in health care in Australia^12^ and the implementation of a $22B per year National Disability Insurance Scheme,^13^ understanding both consumer and citizen preferences for the delivery of health‐care services is paramount.

One method to engage the public in health policy processes, and which is also believed to increase the ecological validity of decisions made and build decision‐making capacity among policymakers and the public, is the use of a Citizens’ Jury.^14^, ^15^, ^16^ A Citizens’ Jury is an active approach^10^ of engaging the public in deliberations on a range of topics across all sectors such as health, education, transport and industry, to craft thoughtful solutions to vexed or entrenched problems.^14^, ^17^ Using this method, a range of formal or informal deliberative processes and literature review material can be presented to Jury members. When adopted in a health‐care context, non‐expert participants consider the realities of health policy development, are exposed to the perspectives and experience of others and reach consensus on recommendations for action.^18^, ^19^, ^20^, ^21^, ^22^, ^23^ Public deliberations, using non‐expert citizens as participants, can make explicit the barriers and facilitators to health‐care policy that are difficult to draw from experts in the field or published research.^24^, ^25^ As such, they balance the dominant interests and perspectives of clinicians and researchers with those of less powerful citizen stakeholders.^7^

Rehabilitation services for people with moderate to severe brain injury in Australia are funded and implemented following government policy. To implement a model of care within a health‐care organization, the facility must first comply with government policy directions, and then clinicians within the organization determine the interventions they perceive should or will be provided.^26^, ^27^ To date, a Citizens’ Jury method has not been used in the context of developing these rehabilitation services, nor in understanding the public's preferences for delivery of specialist brain injury care. The overarching research question addressed by this Citizens’ Jury was as follows: ‘What considerations are important to include in a model of care of brain injury rehabilitation?’ Further specific questions asked of the jury were as follows: Are there circumstances where it is acceptable to not provide rehabilitation to someone with a severe brain injury? Should patients be given a choice over where they are treated, by whom (the type of health professional), and what treatments they are offered? Should family members be considered as equal partners with the patients admitted? How can information about rehabilitation be provided and more easily communicated to people with a severe brain injury and their family? These questions were selected by the authors following review of the literature and gaps identified^2^, ^28^, ^29^, ^30^, ^31^ as well as through anecdotal discussions with therapists and hospital executives engaged in delivering brain injury services at conference meetings. The aims of this study were to (a) identify key themes from jury deliberations, (b) provide juror responses to the four questions posed and (c) formulate citizen‐based recommendations for the delivery of brain injury rehabilitation services, taking into account the context of finite health budgets and challenges to working with people with brain injuries, which would improve acceptability and usefulness of rehabilitation to both the general public and to future service users.

## 2 METHOD

A Citizens’ Jury was conducted and is reported in accordance with typically used guidelines.^14^, ^17^, ^25^, ^32^, ^33^ Citizens’ Juries, as with juries in a court of law, are based on the premise that a random sample of the population may hear evidence on a topic and then undertake deliberations that are representative of the conscience, intelligence and preferences of the general public.^17^, ^18^, ^19^, ^32^, ^34^ Citizens’ Juries consist of a sample of people recruited to represent our diverse community, who are convened to hear from a variety of expert ‘witnesses’ and who present a range of perspectives on a particular issue which, in this case, was identified through informal deliberative processes and literature review. The Citizens’ Jury then engages in deliberations among themselves and, ultimately, provides a ‘verdict’ on the issue at hand. In this case, the verdict is a set of findings (key themes), and recommendations for the field. La Trobe University and Alfred Health Human Research Ethics Committees approved this study, and all jurors provided written, informed consent before data collection commenced.

### 2.1 Participants

An experienced Citizens’ Jury facilitator, with no prior experience of brain injury or rehabilitation and who declared no prior assumptions regarding the topic at commencement, was employed to run the event.^17^

#### 2.1.1 Jurors

Using stratified random sampling, jurors were recruited by an independent recruitment company from a database of landline telephone numbers registered in metropolitan Melbourne, Australia (approx. 4.9 million people). People who were aged over 18 years were randomly telephoned to determine eligibility which included having no prior experience with themselves, a family member or close friend having a brain injury, or working in fields that provided them with some knowledge of brain injury. This was to ensure that jurors had no pre‐conceived biases about the delivery of brain injury services based on their personal circumstances.^17^ Facilitation of the citizen's jury additionally sought to support open dialogue, disclosure of biases and presentation of multiple viewpoints of the complex issue in an effort to support critical detachment of jurors.^16^ A list of 30 potential participants who varied in age, gender, employment status and residential suburb (as an indicator of socioeconomic status) was provided to the research team, who then telephoned each potential participant to determine availability for the 12‐place jury (plus two ‘stand‐by’ jurors). Jurors were selected from the list of 30 by the research team based on their demographics, with the aim of including a broad cross section of the community.^17^ Participants were paid an honorarium of $250AUD.

#### 2.1.2 Witnesses

Seven specialists in the areas of acquired brain injury and rehabilitation were identified from the networks of the research team to appear as witnesses. These witnesses were invited to make 10‐minute presentations to the jury regarding pre‐identified topics pertinent to the delivery of brain injury rehabilitation services. Details of the witnesses and the information they presented are in Table 1. Consistent with Citizens’ Jury methodology, these presentations were followed by juror discussions with each witness including some facilitated question and answer formats as well as small group discussions.^15^, ^17^

**Table 1:** Witness presentations delivered during the Citizens’ Jury

Witness	Content overview
University researcher focusing on brain injury	Peter presented an overview of the health, personal, financial and societal impact of brain injury. He also summarized the current Australian rehabilitation system and the evidence for the effectiveness of common brain injury rehabilitation interventions
Funding model expert from a private consulting firm	Therese presented evidence on funding policies, insurance and health‐care decision making, sharing her knowledge of government, community sector organizations and disability insurance schemes and the policies which shape the provision of rehabilitation in this context
Representative of a brain injury peak body	Nick presented his own story of brain injury that began when in 1996 he was involved in a bicycle vs car accident and suffered a brain injury. Nick is now the CEO of an organization representing the interests of, and advocating for, people with brain injury. Nick also presented on the inequities experienced by brain injury survivors across the country
Mother and carer of a person with brain injury	Cheryl presented her family's story that began when, at the age of 12 y old, her son Jonathan was involved in a car accident and suffered a severe traumatic brain injury. Jonathan was in a coma for six weeks and given very little chance of survival. Cheryl outlined the family role, the burden of advocacy and the important role that hope has played in Jonathan's lifelong rehabilitation.
University researchers focusing on consumer and health‐care service partnerships	Sophie and Nerida outlined the value of involving consumers in the planning and delivery of health care. They explained the various ways hospitals can work in partnership with service users and their families, and the challenges faced in ensuring a service meets the needs of all potential users
University researcher specializing in knowledge translation	Kate presented evidence on the uncertainty in health care and challenges in providing evidence‐based care, sharing evidence of the struggle between research and clinical care.
University researcher specializing in delivery of long‐term care and resources for people living with brain injury	Libby presented evidence on community‐based neurotrauma rehabilitation success and the importance of choice in post‐hospital living arrangements. She explained the short‐term nature of current service provision, the impact that funding has on long‐term access to services and housing, and the personal impact this has on people likely to access the brain injury rehabilitation services being delivered

### 2.2 Data collection

The Citizens’ Jury was held over two consecutive days (total 14 hours) on the grounds of a rehabilitation hospital with specialist brain injury unit in Melbourne, Australia. Jurors were welcomed to the event by the facilitator and lead researcher. Initially, the jury were provided with evidence from the witnesses. In a process supported by the jury facilitator, jurors then had the opportunity to scrutinize and discuss the information and deliberate together to form a view on the research questions.^17^, ^20^ All jury discussions were audiotaped. At the conclusion of the two days, the jurors presented their findings as a set of recommendations to the researchers and invited hospital executives and health‐care professionals working in traumatic brain injury service provision from across the state of Victoria (population of approx. 6.5 million).

### 2.3 Data analysis

Following the final witness presentations, the experienced Citizens’ Jury facilitator^17^ supported deliberations and discussions designed to help jurors focus on the principles they identified as important in determining priorities for brain injury rehabilitation. Emerging issues and points of contention were explored until consensus was reached. This consensus was distilled by the jurors as a set of responses to the four research questions, and a final set of overall recommendations.

After the Citizens’ Jury, the audiotapes of the jury discussions were transcribed verbatim to ensure that all information was accurately captured. Two authors also acted as scribes across the two days (KL and NH), noting key ideas during all discussions. These were analysed alongside the transcripts, increasing the depth and credibility of data collection and triangulating data sources, particularly in relation to theme development.^35^ The research team then coded and thematically analysed the discussions of the jurors. The analysis utilized a realist approach to discourse analysis, particularly thematic analysis,^35^ and utilized the deductive framework advocated by Scott and colleagues.^14^

## 3 RESULTS

The results are arranged in keeping with the presentation of many Citizens’ Jury findings; initially, information about the participants is provided, followed by presentation of key themes as identified by the researchers, and finally presentation of the jurors’ responses to the questions posed for the event, and their recommendations for future policy in the field.

### 3.1 Jurors

As intended through the random telephone selection procedure, the characteristics of the 12 jurors varied widely. Jurors rated their level of involvement in their local community (1 = not involved at all, 5 = extremely involved) prior to commencement. No jurors rated themselves as being extremely involved and 3 rated themselves as not being involved at all, score mean = 2.6 (SD 1.4). Variability in juror age groupings, gender, education levels, employment and income is presented in Table 2.

**Table 2:** Characteristics of jurors (participants)

Characteristic	(n = 12)
Age in y, mean (SD)	40 (19)
Age groupings, number (%)	
Under 25 y	3 (25)
25‐35 y	2 (17)
36‐45 y	1 (8)
46‐55 y	3 (25)
56‐64 y	1 (8)
65 y and older	2 (17)
Sex, number male (%)	5 (42)
Education, number (%)	
High school only	2 (17)
Technical college / trade certificate	2 (17)
University	6 (50)
Post‐graduate qualification	2 (17)
Employment, number (%)	
Unemployed	1 (8)
Student	2 (17)
Retired	2 (17)
Full‐time employment	6 (50)
Part‐time employment	1 (8)
Home ownership, number (%)	
Rent	5 (42)
Mortgage	4 (33)
Own	3 (25)
Home environment, number (%)	
House	8 (67)
Unit / townhome	3 (25)
Apartment / flat	1 (8)

### 3.2 Themes developed from jury discussions

Seven themes were developed by the research team based on all data collected. A summary of these themes is presented below together with illustrative quotes, and Table 3 presents each of these themes with identified subthemes and brief descriptions.

**Table 3:** Themes highlighted during the Citizens’ Jury

Theme: Consumer focus	Categories of coded statements
Subtheme: Individualized and tailored rehabilitation	Flexibility in timing of rehabilitation. Tailoring rehabilitation to patients’ current and future requirements. Choice (of rehabilitation service and of treating clinicians).
Subtheme: Patient‐directed goal setting towards the patient‐selected future	Patient‐directed goal setting to increase accountability and engagement in rehabilitation. Patients should identify their own goals—labelling goals as ‘unrealistic’ suggests that the clinical team involved have not tried to understand the goals of a patient or their family. Commitment to working alongside the patient and their family to work towards their goals.
Subtheme: Retaining hope	Acknowledgement the person that the patient was before their brain injury and their hopes for returning to that pre‐injury life.

Theme: Long‐term	Categories of coded statements
Subtheme: Rehabilitation for life	Treatment plans will alter between rehabilitation settings. Rehabilitation is not linear, and plateaus occur. Family plays a critical role.
Subtheme: Prognosticating is often inaccurate	Definitive prognosis in the early stage of rehabilitation is often incorrect and may reduce hope. Ask what information would be most helpful for the family and explore different ways of communicating prognosis pathways to meet the individual needs of the patient and their family.

Theme: Equitable access	Categories of coded statements
Subtheme: Government funding	All rehabilitation is funded by government in one form or another (state and Commonwealth health, Workcover, Transport Accident Commission, National Disability Insurance Scheme) and therefore, there should be equitable across funding schemes.
Subtheme: Rehabilitation should be available for all Australians	Models of care should account for access for people living in outer metropolitan, regional and remote areas, and those who are from socially marginalized groups.

Theme: Family‐friendly	Categories of coded statements
Subtheme: Families as partners in rehabilitation, patient advocates and case managers	Patients should determine how much family involvement there is at any time point along the continuum of recovery. Families should be provided support and acknowledgement of the trauma which family members experience as a result of the ABI. Counselling for families should be inherent within the model of care; financial counselling may also be warranted, depending on the impact that an acquired brain injury has on the family's financial circumstances.
Subtheme: Fear of the unknown ‐ the novice family member	The general public do not understand brain injury and so access to accurate information and education is critical. Communication and education about rehabilitation approaches is important as the family will be making decisions regarding rehabilitation as well as providing some of the therapies.
Subtheme: Supporting possible futures	Families should not be seen as the default carer during discharge planning by hospital staff. Discharge planning should include support for families to explore access to community carer supports early in their rehabilitation stay to ensure a continuity of not only the patient's care, but the family's ability to provide care.

Theme: Advocacy	Categories of coded statements
Subtheme: Advocacy	All patients should be appointed an advocate who retains this role throughout their rehabilitation. Knowledge of hospital and rehabilitation processes, the impact and possible outcomes of the ABI, and of the process of care, is lacking. Therefore, families need an advocate within the hospital system to ensure that they are able to understand, process and be an active partner in their family member's care

Theme: Public awareness	Categories of coded statements
Subtheme: Raising awareness: Prevention	Awareness information should be made relevant and prevention campaigns should target those most at risk (young males). The general public should become aware of the devastating effects of brain injury and the cost to the community
Subtheme: Raising awareness: Community reintegration	The profile of existing support services should be improved, to both assist in raising funds and to ensure families may locate these services when they need to. Increased awareness may assist with the public acceptance of reintegration of patients recovering from brain injuries into their local and social networks.
Subtheme: One‐stop Information Shop	A government‐funded information and resource source (internet) should be made freely available. Australian government information should include synthesis of international research and should include a quality of information rating to ensure only correct information is provided to the public

Theme: Practical service delivery	Categories of coded statements
Subtheme: Recruitment of skilled staff	Assumptions are made that all staff are highly trained in brain injury rehabilitation Research evidence is not always translated into practice by staff
Subtheme: Daily rehabilitation	Rehabilitation services should be available every day (including weekends) to support recovery.
Subtheme: Physical environment	Given the length of time patients receive rehabilitation, the stressful and difficult nature of this time for the patient and family, the environment is very important. An environment that is modern and spacious with lots of light is important. There needs to be indoor and outdoor spaces where family can be by themselves, or out of the patient's room with the patient.

#### 3.2.1 Consumer focus

The jury was unanimous that brain injury rehabilitation should be tailored to the needs of each patient (current and future). They recommended that flexibility should be built into the health‐care system to allow patients to access facilities and resources at the time most beneficial to them:

It's acceptable to say, ‘No, this person isn't a candidate for rehabilitation right now’ but that doesn't mean that in a bit of time they won't benefit [from rehabilitation] – Juror 9. (female)

The jurors felt strongly that models of brain injury rehabilitation should build and support hope after a catastrophic ABI, rather than give a prognosis early which may devastate the family unnecessarily:

After hearing [Witness‐ mother], I definitely think families should be given hope. Doctors are just people. I mean, doctors may not actually know‐ Juror 3. (male)

The jurors perceived that the current system was not always consumer‐focused (patient‐directed) and certainly does not permit choice for patients or families:

If people are going to put a lot of effort and time into their rehabilitation then they should have some control over how it's run‐ Juror 7. (male)

#### 3.2.2 Long term

The jurors considered it important that a person with a brain injury retains an average life expectancy and, with injury often sustained early in life, the majority of people living with a brain injury are under 65 years of age. In response, they discussed that rehabilitation should be lifelong:

Maybe a person isn't ready for rehabilitation at a certain time, but maybe that would ‐ they've got their whole lifetime to deal with this injury. Maybe that will change down the track, because it is such a long process‐ Juror 9. (female)

Lifelong reassessment of situation and responding to that. If rehab wasn't provided, continually reassessing, and providing re‐entry options‐ Juror 4. (female)

#### 3.2.3 Equitable access

The jury felt strongly that there needs to be equitable access to services regardless of the government funding stream. The lack of consistent access to services across different funding streams did not seem reasonable to jurors.

I think there needs to be a blanket [referral]. It can't be up to the doctor to decide who can receive [rehabilitation. I’m] saying that everybody should get a guarantee [to receive rehabilitation]‐ Juror 5. (male)

#### 3.2.4 Family‐friendly

Jurors qualified that priority should always be given to supporting the person with a brain injury's wishes when known, although the needs of the whole family and ensuring that the model of care remains family‐friendly are critical:

I think she [Witness‐ mother] should actually be given a choice in how and where they're treated – Juror 11. (female)

Families should be equal partners in the rehabilitation – Juror 3. (male)

Specific recommendations were that the family should be given as much information as possible during the initial stages and support and acknowledgement of the trauma which family members have experienced as a result of the brain injury. Family members may require services and support, in the same way that the patient receives funded assistance. The jury felt strongly that communication of the rehabilitation approaches, collaboration for discharge planning and education of the family who will be providing some of the rehabilitation is integral to achieve a good outcome:

At the moment, the system forces the family into the carer mode…, even though that may destroy individuals and the family unit as a whole‐ Juror 12. (female)

#### 3.2.5 Advocacy

The importance of having access to an advocate throughout rehabilitation was highlighted as a significant need for a person with a brain injury.

It's all the more important if there's no family that there's some ‘continuity of concern’ – Juror 11. (female)

#### 3.2.6 Public awareness

The jury acknowledged their own increased knowledge of brain injury rehabilitation through their participation in the Citizens’ Jury. They felt the information would be more broadly relevant to the general community and suggested targeted campaigns about prevention for those most at risk (young males) and generally raising awareness of the effects of brain injury and the cost to the community. The jury felt public education programmes could raise the profile of existing support services and contribute to the awareness of reintegration of patients recovering from brain injuries into their local and social networks. The jury also considered the need for an online ‘one‐stop shop’ for information and resource links about TBI, one which synthesized the information available internationally, with some form of quality control to ensure that only correct information is consumed.

I just think … people ‐ they don't have access to information … it would be good if there was just one person that's just dedicated to you … and can tell you all the resources out there that are applicable to you, rather than you having to go out and source them from different [government] departments, which can be really confusing for a regular person Juror 9. (female)

#### 3.2.7 Practicalities in service delivery

The final theme extracted related to pragmatic issues in delivering brain injury services. The jurors acknowledged that if they were to put themselves into the same position as families entering the rehabilitation system for the first time, they would *assume* that all treatments and rehabilitation were provided by highly skilled staff. Juror's expressed disappointment that despite an available evidence base of proven treatments and approaches, staff may not provide these to all potentially appropriate people. It was acknowledged that reasons underpinning staff decision making with regard to implementation of evidence into practice in this complex health‐care area merits further research.

A family comes into this situation, a client comes into this situation, they don't know anything about the qualifications etcetera [of the staff]. There should being [sic] a supervisor …that is able to keep it [rehabilitation] in an evidence‐based direction‐ Juror 1. (male)

Jurors also expressed disappointment that people with brain injury may remain in hospital on weekends but do not routinely receive an active rehabilitation programme over this period and thought that rehabilitation should be available every day. Finally, the jurors had received a tour of the modern and spacious brain injury rehabilitation unit that hosted the event prior to commencing the two‐day jury. This had an impact on the jurors who commented on the value of this modern environment and highlighted the need for funding to update and modernize all brain injury inpatient rehabilitation facilities to be aesthetically pleasant to live in.

Rehabilitation facilities need to be atheistically pleasing… to live in and if the ABI units were built some time ago, further funding is needed to update them – Juror 3. (male)

### 3.3 Jury response to questions posed and recommendations

At the end of the two days, the jury was asked to summarize their responses to the four questions posed at the outset of the event, and their consensus statements to these questions are presented in Table 4. Finally, the verdict from the jury which is presented as a set of recommendations concerning ‘What considerations are important to include in a model of care of brain injury rehabilitation?’ is presented in Table 5.

**Table 4:** Questions posed to the Citizens’ Jury and their responses for brain injury rehabilitation

Posed question	Response from Citizens’ Jury
Are there circumstances where it is acceptable to not provide rehabilitation to someone with a severe brain injury?	Qualified agreement, but the decision should be related to quality of life considerations, not funding availability
Should patients be given a choice over where they are treated, by whom (the type of health professional), and what treatments they are offered?	Unanimous agreement
Should family members be considered as equal partners with the patients admitted?	This depends on family circumstances. When this is the case (affirmative), resources funded by the hospital should certainly be provided to family; possibly on a trial basis with reviews built in
How can information about rehabilitation be provided and more easily communicated to people with a severe brain injury and their family?	Information should be available in several formats, both at a community level, the level of the hospital / service, as well as at an individual patient level. The personal stories (person living with a brain injury; family living with a person with a brain injury) heard within the Citizens’ Jury were considered to be extremely valuable

**Table 5:** Juror recommendations for model of care of brain injury

Recommendation	Description
Flexibility	Patients should be able to access facilities and resources at the time most beneficial to their circumstances, and of their choice
Family involvement	Families should be included as partners in the rehabilitation and supported as much as possible
High quality staff	Recruitment and retention of highly skilled staff is critical. Patients and their families should be able to be certain that the staff they are working with are suitably skilled and understand evidence‐based practice
Weekend rehabilitation	Patients and their families should be able to receive specialist rehabilitation therapies on the weekend
Modern rehabilitation facilities	The jury recommended further funding towards updating and modernizing inpatient rehabilitation facilities to be aesthetically pleasing and pleasant to live in
Brain injury prevention	Traumatic brain injury prevention education should be provided to target populations
Community appreciation	The general public should be made aware of how they can support community members living with an acquired brain injury and their family members
Advocacy	The appointment of a knowledgeable professional as a ‘Brain Injury Rehabilitation Ombudsman’ would allow patients with an ABI to have a single point of contact for advice about best evidence, to raise concerns about their own rehabilitation and to seek support for family members who are unsure about brain injury or the services they are receiving

## 4 DISCUSSION

This unique Citizen's Jury investigated ‘What considerations are important to include in a model of care of brain injury rehabilitation?’ The research team identified seven themes from the juror discussions and deliberation which were summarized as: being consumer‐focused; that rehabilitation is viewed as a long‐term undertaking; equitable access to services irrespective of funding source; family as programme partners; need for advocacy; raising public awareness around prevention and community reintegration; and pragmatics issues in delivering a brain injury service. The jury answered the four research questions posed and determined that in some cases, it may be acceptable to not provide rehabilitation to someone with a severe brain injury, that patients should be given a choice over where they are treated, by whom and the type of treatments offered, that in many cases the family members be considered as equal partners with the patients admitted and that brain injury information should be available for patients and families in several formats to increase communication. Finally, the jurors delivered eight recommendations for policy maker in the provision of brain injury rehabilitation, which closely reflect the themes as developed by the research team from their discussions as well as the development of answers to the four specific questions. These were as follows: flexibility and choice in service provision, importance of family involvement as equal partners, as well as the need for high quality rehabilitation staff, 7‐day‐per‐week rehabilitation, brain injury prevention campaigns for the public, community appreciation of living with brain injury, on‐going advocacy and modern facilities.

Despite their lack of any experience in this field, jurors demonstrated an understanding of the importance of evidence‐based practice and finite health budgets and were able to make empathetic recommendations. However, many of the initiatives raised do require resourcing which is not readily resolved. For example, Table 5 presents the Jurors’ view that patients should be able to access rehabilitation at any time post‐injury as not everyone is able to take best advantage of rehabilitation early in their recovery and funding provided at this time may not be most efficient. Therefore, further research to better understand the clinical efficiencies and outcomes of differing timing for offering rehabilitation services is required.^36^ Jurors appeared to recognize the hardships that family and individuals with brain injury face and reflected on how they would want to be treated if they themselves or their family were in the same situation. Research with consumers of specialist brain injury services supports the jurors’ recommendations. For example, Canadian researchers LeFebvre, Pelchat, Swaine, Gelinas and Levert^28^ used semi‐structured interviews to investigate the experiences of eight adults who had sustained a brain injury, their families and the clinicians involved in their care. Similar to the current study, LeFebvre and colleagues reported on the importance of having the best, and sufficient, human resources during rehabilitation and proposed that a lack of staffing leads to ‘exhaustion’ among clinicians which then compromises quality of care for patients. The findings of Hartwell et al^37^ and Muus, Cogan, Offutt and Medalen^38^ also supported the current study recommendations, outlining the importance of having brain injury advocates, of adequate knowledge / information and of adequate financial resourcing at the state level when delivering brain injury rehabilitation. The jurors reported that an ombudsman could be appointed for brain injury rehabilitation, which would be consistent with other complex areas of care such as the appointment of the Mental Health Complaints Commissioner, a role which was created in 2014 in Victoria, Australia (where this research was conducted). The ombudsman would be impartial and act in the interests of the person with brain injury and their family in navigating the heath service, and identify and communicate systemic change to maximize health service provision.

While our findings may be challenging and confronting to both clinicians and hospital administrators, some of the themes generated and recommendations made by our critically detached jurors mirror the concerns highlighted in other research investigating consumer preferences and experiences in brain injury rehabilitation.^28^, ^38^, ^39^ These included the importance of hope in recovery which has been reported by both clinicians^40^, ^41^ and people with lived experience,^42^ increasing public awareness of brain injury^34^ and the need to involve family as partners in rehabilitation.^30^, ^31^ It is acknowledged, however, that none of these issues are straightforward. For example, the idea of supporting hope in the early stages of recovery presents an ethical dilemma for clinicians who need to balance the harsh realism of presenting likely outcomes with the faint possibility of better‐than‐expected recovery. None the less, the jurors in this research were very specific regarding maintaining hope.

While several of the findings as noted above are common across the brain injury literature, other recommendations are unique to our study such as the need for service flexibility so patients can access services at the right time in their recovery, and the importance of the physical environment. This may be because a Citizens’ Jury approach meant that jurors themselves deliberated, debated and wrote their own recommendations, rather than the researchers. Variations between findings may also have been because of the lack of experience in brain injury by jurors in the current study. Three of the 12 jurors were people who do not normally participate in community engagement activities, representing views of an underrepresented group. This highlights the importance of understanding *both* the perspectives and preferences of those with lived experience and of citizens when planning health‐care services.

While the main strength of using a Citizens’ Jury is the informed, democratic and deliberative process undertaken to engage members of the public.^14^ there are several limitations that need to be considered. For financial reasons, our sample population was limited to metropolitan Melbourne, and therefore, findings may not generalize to rural or to national brain injury services. A stratified random sampling was used to select jurors to maximize the diversity of the sample; however, we acknowledge that because the external recruiting agency used electoral data, we were unable to include ethnicity in the sampling frame which may have implications for the findings drawn by our jury. A second limitation of the design may have been the length of time jurors spent together. Mitton and colleagues’ review of public participation methods^43^ suggested that one‐off initiatives, such as occurred in this jury, may fail develop meaningful communication and trust between participants. While the facilitator dedicated the initial two hours of this Citizens’ Jury to trust‐building exercises, it is plausible that engagement may have been higher if jurors met for a longer period of time, or for a similar number of hours but over a number of weeks rather than consecutive days. Another consideration is the development and usefulness of the outcome recommendations. The jurors in this study reported feeling rushed in the development of responses to the questions and recommendations and also being concerned that their findings may not be used by health services and policymakers. Again, having a longer time together may have led to more synthesized findings; however, it remains a strength of our design that this Citizens’ Jury was attached to a health service who were receptive to the jurors presenting their findings directly to hospital executives and health‐care professionals. Abelson et al^20^ suggest that having such explicit links to policymakers leads to greater knowledge exchange from public participation methods. Finally, there is an acknowledged barrier to using research findings to change policy in Australia^44^ particularly from only one source of public opinion. Given the complexity of the needs expressed by members of the jury, we recommend further research be undertaken to better understand public opinion regarding brain injury rehabilitation service delivery and that such research be undertaken in collaboration with policymakers to facilitate possible future change.^45^

## 5 CONCLUSION

Like Citizens’ Juries held in other health areas,^21^, ^22^, ^23^ our research findings demonstrate this method can be effectively used to inform decision making when delivering complex and expensive health services such as those offered in brain injury rehabilitation. The citizens who participated in this study recommended that rehabilitation for adults after brain injury include careful consideration of flexibility for how services are offered, family involvement, recruitment and retention of highly skilled staff, and provision of seven‐day rehabilitation. Brain injury services across Australia, and also internationally, can map these jury recommendations against local services provided and clinician preferences for brain injury rehabilitation to reflect on the approach taken and adjustments that may enhance service quality. On‐going research to ensure rehabilitation services are underpinned by evidence, and offer the best treatments available, remains vital.

## CONFLICT OF INTEREST

All authors declare no conflicts of interest.

## AUTHORS CONTRIBUTIONS

NL received the funding and designed the study designed the study with AH and CU. Data collection conducted by all authors. NL, MC and CU drafted the manuscript. All authors critically appraised and edited drafts of the manuscript. All authors read and approved the final manuscript.

## Funding information

The study was supported by a local grant from La Trobe University (College of Science, Health and Engineering); National Heart Foundation of Australia supports the following authors: NAL GNT 102055; CE GNT 101177.

## ETHICAL APPROVAL

The Alfred Health and La Trobe University Human Research Ethics Committees approved this study [HREC 401‐14]. All participants gave written informed consent before data collection began.

## Data Availability

Due to confidentiality and the nature of the consent obtained, the transcripts of the Citizens’ Jury cannot be shared. For further information relating to this data, or to obtain the data from the Expert Witnesses and final recommendations, which can both be shared, please contact the primary author.
